# Bayesian Hierarchical Clustering for Studying Cancer Gene Expression Data with Unknown Statistics

**DOI:** 10.1371/journal.pone.0075748

**Published:** 2013-10-23

**Authors:** Korsuk Sirinukunwattana, Richard S. Savage, Muhammad F. Bari, David R. J. Snead, Nasir M. Rajpoot

**Affiliations:** 1 Department of Computer Science, The University of Warwick, Coventry, United Kingdom; 2 Warwick Systems Biology Centre, The University of Warwick, Coventry, United Kingdom; 3 Department of Pathology, University Hospitals Coventry & Warwickshire, Coventry, United Kingdom; 4 Divisions of Reproduction and Metabolic & Vascular Health, Warwick Medical School, Coventry, United Kingdom; 5 Department of Computer Science and Engineering, Qatar University, Doha, Qatar; University of Turin, Italy

## Abstract

Clustering analysis is an important tool in studying gene expression data. The Bayesian hierarchical clustering (BHC) algorithm can automatically infer the number of clusters and uses Bayesian model selection to improve clustering quality. In this paper, we present an extension of the BHC algorithm. Our Gaussian BHC (GBHC) algorithm represents data as a mixture of Gaussian distributions. It uses normal-gamma distribution as a conjugate prior on the mean and precision of each of the Gaussian components. We tested GBHC over 11 cancer and 3 synthetic datasets. The results on cancer datasets show that in sample clustering, GBHC on average produces a clustering partition that is more concordant with the ground truth than those obtained from other commonly used algorithms. Furthermore, GBHC frequently infers the number of clusters that is often close to the ground truth. In gene clustering, GBHC also produces a clustering partition that is more biologically plausible than several other state-of-the-art methods. This suggests GBHC as an alternative tool for studying gene expression data.

The implementation of GBHC is available at https://sites.google.com/site/gaussianbhc/

## Introduction

Clustering analysis is an important tool in studying genomic data such as gene expression profiles and can be used to infer biological function and regulation of genes. Eisen *et al.* [1] found that in yeast *S. cerevisiae*, genes that are clustered together often share similar biological function or are co-regulated, leading to the recognition that genes in the same cluster can be functionally related or regulated by a common set of transcription factors. It has been shown in the literature that biological function of a cluster can be inferred from ontology annotation of its genes [2], and biological function of an uncharacterized gene can also be inferred from the knowledge of genes in its cluster [3], [4]. Moreover, in modern medical research, clustering analysis has been used to identify disease subtypes based on genetic variation [5], [6], and to identify a gene expression signature that can be used as a prognostic marker for known disease subtypes [7]–[9]. This aids stratification of patients for personalized medicine.

Numerous commonly used clustering algorithms have a significant limitation in that they rely on *ad hoc* methods to identify the number of clusters within the data. In hierarchical clustering algorithms [10]–[12], for example, identifying the number of clusters mainly depends on visual identification, whereas the number of clusters is required as an input to other algorithms such as 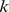-means [13] and self-organizing map [14]. Furthermore, many clustering algorithms require the choice of a distance metric to indicate the strength of similarity/dissimilarity between data points or clusters. However, there is little systematic guidance about how to choose a metric for data such as gene expression measurements that reflects reasonably well the relationship between data. Often, it is difficult to define the relationship, especially in high-dimensional space. Two common choices of metrics in gene clustering analysis literature are Euclidean distance and Pearson correlation coefficient [15]. However, Euclidean distance is sensitive to scaling and differences in average. Pearson correlation coefficient can only capture linear relationship between data, and it is not robust to outliers and non-Gaussian distribution [16]. Model-based clustering algorithms can address both of these problems. In model-based algorithms, data are represented by a mixture model [17], [18] of parameterized distributions, in which each component represents a different cluster. The problems of how to identify the number of clusters and the distance metric can therefore be cast as a model selection problem - how to choose a statistical model that best describes the data.

Bayesian hierarchical clustering (BHC) [19], [20] is a model-based clustering algorithm based on the Dirichlet process mixture model (DPM) [18], [21], [22]. It has strong advantages over other model-based approaches. First, it produces a hierarchical clustering structure which is more informative than a flat one. Second, it uses Bayesian model selection to determine the hierarchical structure, rather than an *ad hoc* distance metric, thereby increasing the quality of resulting clusters. Multinomial BHC (MBHC) [23] represents the data in each mixture component as a product of multinomial likelihoods, subject to a Dirichlet prior, and has been shown to produce higher dendrogram purity and more biologically meaningful clusters than other commonly used algorithms for the *Arabidopsis thaliana* microarray dataset [23]. However, by using multinomial likelihoods, the algorithm requires a categorical approximation of a continuous variable. This may not, therefore, fully capture the underlying structure of continuous gene expression data. Gaussian likelihoods are an obvious alternative here, as they do not require data approximation and have been used for describing gene expression data in many clustering analyses. Previous work on expression datasets of ovary and yeast cell cycle show that model-based clustering algorithms that use finite Gaussian mixture model produce comparable quality clusters to a leading heuristic clustering algorithm, even if the data do not totally satisfy Gaussian mixture assumption [24]. In a comparative study of clustering algorithms for cancer gene expression data, given the actual number of clusters, finite Gaussian model approach is the leader in assigning data to the correct cluster [25]. Rasmussen *et al.* [26] propose a model-based clustering algorithm with infinite Gaussian mixture model to study Rosetta compendium of expression profiles of *S. cerevisiaie*, and find that clustering results not only confirm previously published clustering analyses but also reveal finer clustering level that are novel and biologically consistent.

In this paper, we propose an extension of the BHC algorithm for gene expression data which we term as the Gaussian BHC (GBHC). GBHC offers several advantages over other clustering algorithms: first, it assumes an infinite Gaussian mixture model for gene expression data, which has been shown to be biologically plausible in literature [24]–[26]; second, it employs the mixture model in a Bayesian framework to perform a model-based hierarchical clustering of gene expression data revealing hierarchical structure present in the data; third, it infers the number of clusters automatically from the data; and fourth, it uses the Gaussian mixture assumption to describe the data and uses a normal-gamma distribution as a conjugate prior on unknown means and precisions of the Gaussian likelihoods. We introduce two variants of GBHC: one with hyperparameter optimization over the whole tree (GBHC-TREE), and another with hyperparameter optimization at every merger (GBHC-NODE). Further, we derive a tractable formulation for speeding up the hyperparameter optimization in case of GBHC-NODE, resulting in a speedup factor of up to 11 over GBHC-TREE. We compare these two algorithms with a range of other clustering methods, performing a study over 3 synthetic datasets and 11 cancer gene expression datasets. The results show that although the data are not very well-represented by a mixture of Gaussian distributions, both variants still improve the clustering quality if the data are normalized and do not have strong correlation between variables. On average, both flavors of our GBHC algorithm produce clustering results which compare favorably to the existing approaches.

## Materials and Methods

### Notations[Table pone-0075748-t006]

**Table pone-0075748-t006:** 

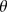	unknown parameters of a probabilistic model component in a mixture model
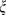	hyperparameters of a prior on 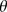
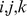	indices
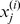	a data value of the 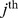 variable from the 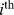 observation
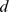	total number of data variables
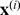	a data point 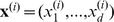
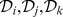	the 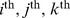 cluster of data points, respectively
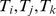	a merger that makes 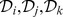 , respectively
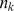	number of data points in 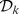
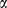	concentration parameter of the Dirichlet process mixture model
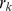	merger probability
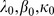	hyperparameters of the marginal likelihood for a Gaussian distribution with a normal-gamma prior
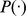	probability
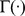	gamma function
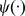	digamma function, defined by 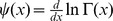
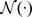	probability density function of a Gaussian distribution
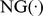	probability density function of a normal-gamma distribution
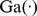	probability density function of a gamma distribution

### Bayesian Hierarchical Clustering Algorithm

**Figure 1:**
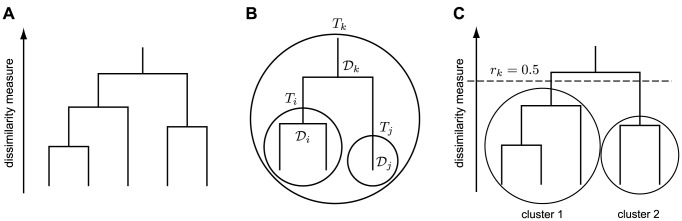
Bayesian hierarchical clustering. A) A dendrogram represents the merger process of BHC. Each vertical line represents a cluster. A horizontal line connecting between any two vertical lines represents the merger of clusters, where its height is related to the dissimilarity measure between the merged clusters. B) A schematic shows datasets 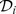 and 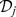 merged into 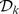, where 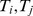, and 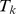 are the associated mergers that make 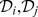, and 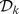, respectively. C) BHC prunes the dendrogram at 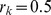, resulting in the final partition.

BHC [19] assumes that data are generated from a mixture model, in which each cluster within the data corresponds to a different distribution component of the model. Suppose that data points 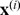 in a cluster 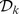 are independently and identically generated from a probabilistic model 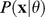 with unknown parameters 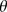, and 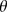 are governed by a prior 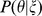 with hyperparameters 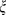. Thus, the marginal likelihood of 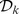 can be expressed by
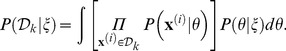
(1)The algorithm initially places each data point into its own trivial cluster and iteratively merges the two most similar clusters, until all the data points are put into a single cluster. This merger process can be represented by a dendrogram (Figure 1A).

The notion of similarity between clusters is related to the probability that they should be merged. This is defined based on Bayesian hypothesis testing as follows. To merge clusters 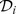 and 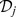 into 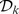 (Figure 1B), BHC considers the null hypothesis 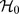: 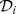 and 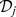 belong to 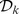 and the alternative hypothesis 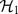: 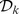 consists of two or more clusters. The probability that 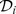 and 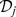 should be merged is calculated via the Bayes' rule:
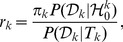
(2)where a marginal likelihood 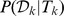 is defined recursively by
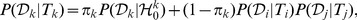
(3)

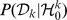
 is a marginal likelihood of 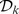 given in Equation (1), and 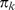 is a prior that 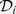 and 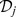 should be merged and is defined recursively by
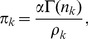
(4)

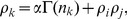
(5)where we set 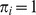 and 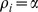 for every initial cluster 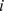. We note that the definition of 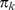 defined here makes Equation (3) an approximation of a marginal likelihood of DPM. Moreover, the value of concentration parameter 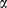 is connected to the expected number of clusters that BHC infers. An increase in 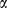 implies an increase in the expected number of clusters.

At 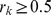, 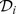 and 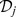 are more likely to belong to the same cluster than at 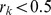. Consequently, we obtain the final number of clusters and partition when all the remaining pairs of merger have 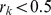 (Figure 1C).

### The Marginal Likelihood for Gaussian Distribution with Unknown Mean and Precision

Consider a dataset in which each observation 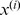 consists of 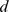 variables, i.e. 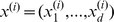. We assume that

**A 1** the dataset is normalized, i.e. it has mean zero and a unit variance;

**A 2** for each observation 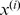, its variables 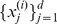 are independent and generated from different Gaussian distributions;

**A 3** the realizations of each variable 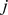, 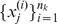 in cluster 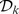 are independent and identically distributed and drawn from Gaussian distribution with unknown mean 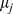 and precision 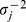, and the prior on 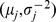 is a normal-gamma distribution with hyperparameter 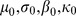.

The probability density function of a Gaussian distribution is defined as
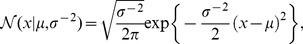
(6)and the probability density function of a normal-gamma distribution is defined as
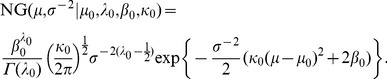
(7)


From the above assumptions, the marginal likelihood of 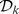 can be expressed as
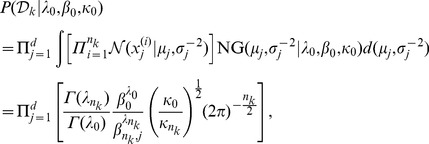
(8)where
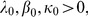
(9)and
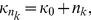
(10)

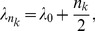
(11)

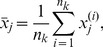
(12)

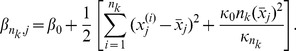
(13)In deriving (8), the hyperparameter 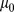 which indicates the mean of parameter 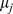 is set to 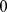 to reflect Assumption A1. Equation (8) is all that is required for 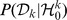 in GBHC.

### Hyperparameter Optimization

GBHC infers the values of hyperparameters 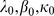 by using the information from 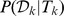 which tells us how well the clustering hierarchy fits the data. This inference can be done via two optimization schemes as follows.

- **Optimization globally over the whole tree (TREE)**. GBHC-TREE finds only one set of optimal hyperparameters 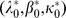 that fits the whole data, and is given by
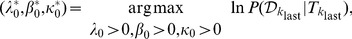
(14)where 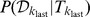 is the marginal likelihood (3) of the final merger in BHC. To learn the optimal hyperparameters in this case is costly since the gradients of 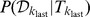 with respect to hyperparameters are analytically intractable, unless the structure of the clustering hierarchy is fixed. (See [19] for more details on optimization of 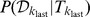 in the case that the clustering hierarchy is fixed.)
- **Optimization at every merger (NODE)**. GBHC-NODE finds optimal hyperparameters 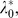 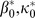 for each merger 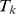 in BHC by performing
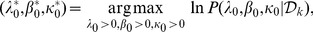
(15)where
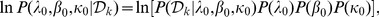
(16)and we assume that
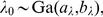
(17)

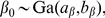
(18)

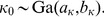
(19)The probability density function of a Gamma distribution is defined by
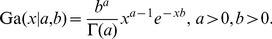
(20)Thus the log-likelihood function in (16) can be written as,
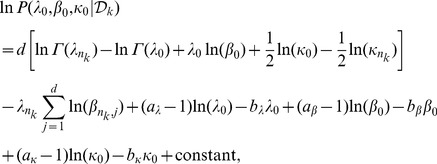
(21)and its gradients with respect to hyperparameters are
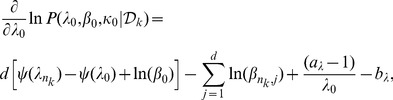
(22)

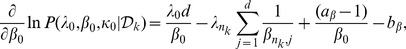
(23)

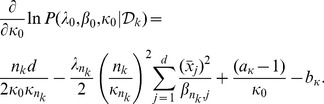
(24)See Section S1 in Material S1 for derivations of Equations (22)–(24). We use weakly informative priors over hyperparameters in Equations (17)–(19), assuming that the data are normalized,
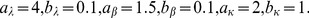
(25)We note that Equation (15) is related to the optimization of 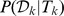, in which approximation 
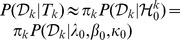
 and the maximization of its posterior distribution is considered. We can see that GBHC-NODE finds the optimal structure of the clustering hierarchy in a single run by searching for the best merger at each level while the hierarchy is constructed. So, it is more time-efficient than GBHC-TREE.

The possible limitation of both optimization schemes is that the optimization objective functions (14),(15) can be non-convex. This will result in GBHC-TREE and GBHC-NODE only finding hyperparameters that are locally optimal. Nevertheless, in our experiments with clustering synthetic data and gene expression data, both schemes have produced promising results.

### Other Clustering Algorithms

We compare GBHC-TREE and GBHC-NODE to other clustering algorithms in Table 1. The algorithms and their similarity/dissimilarity measure will be referred to by the abbreviations given in the table. For instance, APE stands for affinity propagation using negative Euclidean distance. Furthermore, we employ L-methods [27] to infer the number of clusters in AC,AE,CC,CE,KC, and KE, which are the algorithms that require pre-specified number of clusters.

**Table 1:** Clustering algorithm.

Algorithm	Similarity/Dissimilarity Metric	Ability to Infer # Clusters	Reference
**AP**: affinity propagation	**C**: negative one minus Pearson's correlation coefficient; **E**: negative Euclidean distance	yes	[32]
**MBHC**: multinomial BHC	-	yes	[19], [23]
**A**: average-linkage hierarchical clustering	**C**: one minus Pearson's correlation coefficient; **E**: Euclidean distance	no	[10]
**C**: complete-linkage hierarchical clustering	**C**: one minus Pearson's correlation coefficient; **E**: Euclidean distance	no	[11], [12], [33]
**K**: *k*-means	**C**: one minus Pearson's correlation coefficient; **E**: square Euclidean distance	no	[13]

In this work, we implement GBHC-TREE, GBHC-NODE and MBHC in MATLAB. We use AP which is publicly available at the authors' webpage (http://www.psi.toronto.edu/index.php?q=affinity\%20propagation). All the remaining algorithms could be found as MATLAB's built-in functions.

### The Datasets

#### Synthetic Datasets

GBHC-TREE and GBHC-NODE should perform very well if the Assumptions A1–A3 are satisfied. However, real expression data are expected to be not fully satisfied Gaussian mixture assumption, and the correlation between data variables is possible. It is very important to evaluate the performance of GBHC-TREE and GBHC-NODE in comparison to the other clustering algorithms when some of the assumptions are violated. Here, we use synthetic datasets to study GBHC-TREE and GBHC-NODE in three different scenarios as follows (see Section S2 in Material S1 for more details on how the data are generated).

#### Synthetic Dataset1: Mixture of Gaussian Distributions and Independent Data Variables

1000 observations of 10-dimensional random vector are drawn from a mixture of 7 multivariate Gaussian distributions, where each multivariate Gaussian distribution has diagonal covariance matrix. Then the data are normalized.

#### Synthetic Dataset2: Mixture of Gaussian Distributions and Correlated Data Variables

Similar to the first scenario, 1000 observations of 10-dimensional random vector are drawn from a mixture of 7 multivariate Gaussian distributions, but the covariance matrix of each multivariate Gaussian distribution has non-diagonal entries which are non-zero. Then the data are normalized.

#### Synthetic Dataset3: Mixture of Several Distributions

We generate 1000 observations of 10-dimensional random vector from a mixture of 7 different multivariate distributions. For the first 6 multivariate components of a mixture, namely Gaussian, gamma, uniform, student's t, Weibull, and chi-squared distributions, random variables in different dimensions are independent. For the last multivariate component of a mixture which is a Gaussian distribution, there is correlation between random variables in different dimensions. This dataset is normalized prior to the use.

#### Gene Expression Datasets

The performance of all the aforementioned clustering algorithms is assessed through 11 cancer datasets, as described in Table 2. Blood1, Blood2, Bone Marrow, Brain1, Brain2, Colon, Multi-tissue1, Multi-tissue2, Prostate1 are downloaded from http://algorithmics.molgen.mpg.de/Static/Supplements/CompCancer/datasets.htm. These datasets are already filtered according to the protocol described in [25]. We transform every dataset by 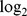 and normalize it before using.

**Table 2:** Dataset detail.

Dataset Name	# Total Samples	# Classes	Classes	# Total Probes	# Remaining Probes
Blood1 [34]	72	2	24 ALL, 48 MLL	12,582	1,081
Blood2 [35]	77	2	58 DLBCL, 19 FL	7,129	798
Bone Marrow [36]	72	2	47 ALL, 25 AML	7,129	1,868
Brain1 [37]	28	2	14 CG, 14 NG	12,625	1,070
Brain2 [38]	42	5	10 MD, 10 Mglio, 10 Rhab, 4 Ncer, 8 PNET	7,129	1,379
Colon [39]	37	2	8 serrated CRC, 29 conventional CRC	22,883	2,202
Lung	16	3	7 NL, 5 LCNEC, 4 SCLC	42,545	2,995
Multi-tissue1 [40]	190	14	11 BR, 10 PR, 11 LU, 11 CRC, 22 LY, 10 ML, 11 BL, 10 UT, 30 LE, 11 RE, 11 PA, 11 OV, 11 ME, 20 CNS	16,063	1,363
Multi-tissue2 [41]	174	10	26 PR, 8 BL, 26 BR, 23 CRC, 12 GA, 11 KI, 7 LI, 27 OV, 6 PA, 28 LU	12,533	1,571
Prostate1 [42]	102	2	50 NP, 52 PR	12,600	339
Prostate2 [43]	19	3	6 benign, 7 primary, 6 metastatic	54,675	1,348

ALL: acute lymphoblastic leukemia; AML: acute myelogenous leukemia; BL: bladder/ureter cancer; BR: breast cancer; CG: classic glioblastoma; CNS: central nervous system; CRC: colorectal cancer; DLBCL: diffuse large B-cell lymphoma; FL: follicular lymphoma; GA: esophageal cancer; KI: kidney cancer; LCNEC: large cell neuroendocrine carcinoma; LE: leukemia; LI: liver cancer; LU: lung cancer; LY: lymphoma; MD: medulloblastoma; ME: pleural mesothelioma; Mglio: malignant glioma; ML: melanoma; MLL: lymphoblastic leukemia with myeloid/lymphoid or mixed-lineage leukemia (MLL) translocations; Ncer: normal cerebella; NG: nonclassic glioblastoma; NL: normal lung tissue; NP: normal prostate tissue; OV: ovarian cancer; PA: pancreatic cancer; PNET: primitive neuroectodermal tumour; PR: prostate cancer; RE: renal cell carcinoma; Rhab: atypical teratoid/rhabdoid tumour; SCLC: small cell lung carcinoma; UT: uterine cancer;

Prostate2 is downloaded from Gene Expression Omnibus (http://www.ncbi.nlm.nih.gov/geo/) (GDS1439). The dataset is transformed by 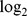 and then filtered by the Wilcoxon rank-sum test at significance level 0.001. The test is carried out between a group of benign and a group of primary and metastatic. The dataset is normalized before using.

Lung is available at Gene Expression Omnibus (GSE44447). The microarray experiment of this data was conducted on Agilent SurePrint G3 Human Gene Expression 8×60 K microarrays (AGilent Technologies, Wokingham, UK), using lung tissues that were ethically approved under the Multicentre Research Ethics Committee (MREC) approval. The experiment was designed to compare the gene expression profiles of two types of closely related high grade neuroendocrine carcinomas, small cacinoma and large cell neuroendocrine carcinoma, which are difficult to classify correctly even for pulmonary pathologists. The raw expression data was processed using R Bioconductor package *limma* (http://www.bioconductor.org/packages/2.10/bioc/html/limma.html), loess and quantiled normalized and corrected for batch effect using *ComBat* (http://www.bu.edu/jlab/wp-assets/ComBat/Abstract.html). We filter this dataset using Wilcoxon rank-sum test for testing the difference between normal and cancer groups at significance level 0.001, and normalize it prior to clustering.

### Clustering Performance Indices

We use two metrics to evaluate the clustering performance: (i) adjusted Rand index (ARI) [28] and, (ii) biological homogeneity index (BHI) [29]. In the clustering of synthetic data, since the true partition of data classes is known, ARI is used as a measure of agreement between clustering partition and the true partition. ARI scores a pair of partitions between 0 and 1, and a higher ARI score indicates higher agreement. We also use ARI in sample clustering experiment of gene expression data.

In gene clustering of gene expression data, we are interested in how biologically meaningful the clustering results are. BHI is used to measure the biological plausibility of gene clustering results generated by an algorithm. It scores a partition between 0 and 1, where a higher score will be assigned to the more biological homogeneous partition based on a reference set of functional classes. In this case, we use Gene Ontology (GO) annotation in Bioconductor package (Section S3, Table S1 in Material S1), while the BHI is calculated using the R package *clValid* [30].

## Results and Discussion

### Synthetic Datasets

**Figure 2:**
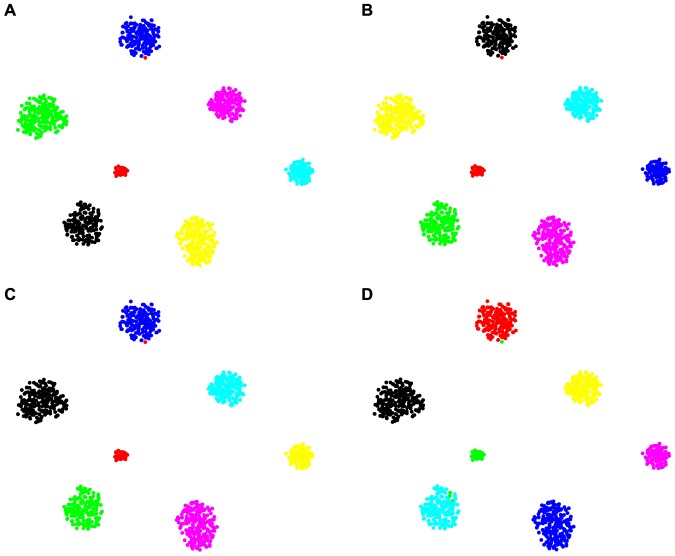
Clustering Results of Synthetic Dataset1. Clusters are represented by different colors or types of marker. A) 7 actual clusters. B) Clustering result produced by GBHC-TREE has 7 clusters. C) Clustering result produced by GBHC-NODE has 7 clusters. D) Clustering result produced by AE has 7 clusters.

**Figure 3:**
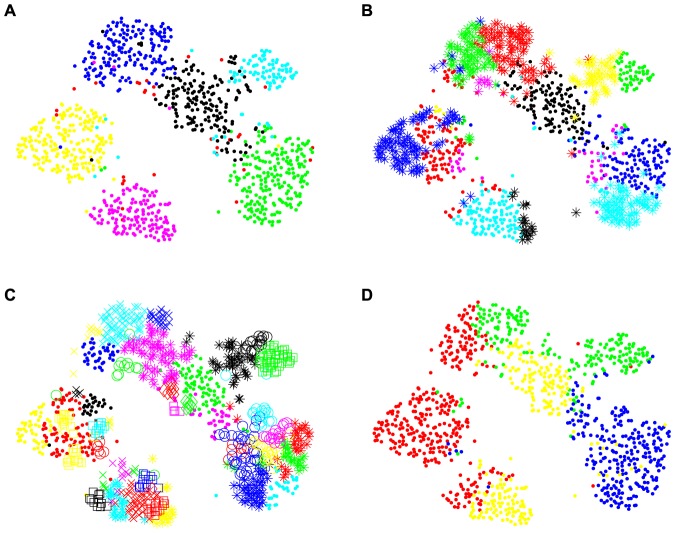
Clustering Results of Synthetic Dataset2. Clusters are represented by different colors or types of marker. A) 7 actual clusters. B) clustering result produced by GBHC-TREE has 14 clusters. C) clustering result produced by GBHC-NODE has 37 clusters. D) clustering result produced by KE has 4 clusters.

**Figure 4:**
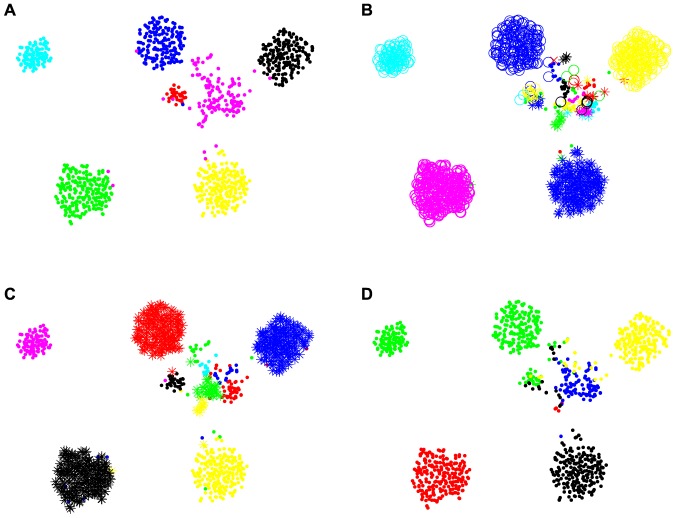
Clustering Results of Synthetic Dataset3. Clusters are represented by different colors or types of marker. A) 7 actual clusters. B) Clustering result produced by GBHC-TREE has 22 clusters. C) Clustering result produced by GBHC-NODE has 12 clusters. D) Clustering result produced by KE has 5 clusters.

ARI scores of clustering algorithms are shown in Table 3, and the numbers of clusters inferred by the algorithms are given in Section S5, Table S2 in Material S1. Details of the experimental setting can also be found in Section S4 in Material S1. For visual inspection of clustering results, we employ a dimension reduction approach called t-Distributed Stochastic Neighbor Embedding (t-SNE) [31] algorithm to reduce the dimension of the original synthetic data into 2-dimensional Euclidean space. t-SNE maps data by preserving the local structure; thus data which are in the same cluster will be placed close by each other in the lower-dimensional space. The visualizations of clustering results are shown in Figures 2, 3, 4.

**Table 3:** Adjusted Rand Index from Synthetic Data Clustering Experiment.

Dataset	APC	APE	GBHC-TREE	GBHC-NODE	MBHC	AC	AE	CC	CE	KC	KE
Synthetic Dataset1	0.317	0.230	**1.000**	**1.000**	0.648	0.938	0.996	0.932	0.954	0.547	0.851
Synthetic Dataset2	0.106	0.095	**0.467**	0.270	0.143	0.324	−0.000	0.132	0.088	0.312	0.413
Synthetic Dataset3	0.295	n/a	0.897	**0.921**	0.643	0.479	0.002	0.710	0.506	0.495	0.750
mean	0.239	0.163	**0.788**	**0.730**	0.478	0.581	0.333	0.592	0.516	0.451	0.671

n/a: not applicable since the algorithm does not converge. Bold number(s) in each dataset highlight(s) the maximum ARI value. Bold underlined numbers highlight the first three highest average ARIs.

#### Synthetic Dataset1: Mixture of Gaussian Distributions and Independent Data Variables

When Assumptions A1–A3 are satisfied, GBHC-TREE and GBHC-NODE outperform the others by correctly infer the membership of data points as well as the number of clusters. On the other hand, there are some minor to high degradation in clustering results from the other algorithms.

#### Synthetic Dataset2: Mixture of Gaussian Distributions and Correlated Data Variables

In the case where Assumption A2 is violated, the performances of GBHC-TREE and GBHC-NODE are highly effected by the correlation between data variables. From Figure 3, we can see that GBHC-TREE and GBHC-NODE infer many sub-clusters of the actual one. The reason is that a bigger cluster of correlated data provides a stronger evidence that the data are not generated from the model underlying GBHC-TREE and GBHC-NODE. Thus, the marginal likelihood (8) gets smaller as the cluster gets bigger, and consequently, GBHC-TREE and GBHC-NODE are in favor of not merging smaller clusters into a bigger one according to Bayes' rule (2). In our experiment, we found that the degradation depends on both the number of correlated pairs of variables and the degree of correlation. The increase in either factor results in the increase in the number of inferred sub-clusters (see Section S5, Tables S3,S4 in Material S1 for details).

#### Synthetic Dataset3: Mixture of Several Distributions

GBHC-TREE and GBHC-NODE are able to recognize all the clusters generated from non-Gaussian distributions even if the distributions are highly deviated from the Gaussian distribution, given that Assumptions A1, A2 are satisfied.

It is apparent that the strong correlation between data variables is the main factor that limits the performance of GBHC-TREE and GBHC-NODE. One could try to transform the data to reduce the correlation between variables before clustering, but one has to bear in mind that the transformation might destroy the meaning of original data variables. Despite the degradation in clustering results, GBHC-TREE and GBHC-NODE still outperforms all the other methods on a whole.

### Gene Expression Datasets

We compare sample clustering and gene clustering performances of GBHC-TREE and GBHC-NODE to those of other algorithms. Note that, in gene clustering, we treat probes as observations and the expression levels across different samples as variables. In sample clustering, on the other way round, samples are treated as observations and the expression levels across different probes are treated as variables.

In sample clustering, Table 4 shows that GBHC-NODE and GBHC-TREE give the highest ARI in 4 datasets (Blood2, Multi-tissue2, Prostate1, Prostate2) and 2 datasets (Bone Marrow, Prostate2), respectively. The other algorithms give the highest ARI in at most 2 datasets. The first three algorithms with the highest mean ARI are GBHC-NODE, GBHC-TREE, and CC. However, there are no significant differences between them (p-value 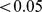; Section S6, Table S5 in Material S1). In terms of accuracy in inferring the number of sample classes (Section S6, Tables S6,S7 in Material S1), the first three algorithms on average are GBHC-TREE, KE, and GBHC-NODE, but there are no significant differences between them (p-value 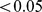; Section S6, Table S8 in Material S1).

**Table 4:** Adjusted Rand index from Sample Clustering Experiment.

Dataset	APC	APE	GBHC-TREE	GBHC-NODE	MBHC	AC	AE	CC	CE	KC	KE
Blood1 [34]	0.246	0.147	0.551	0.474	0.382	0.175	0.206	0.533	0.175	0.576	0.544
Blood2 [35]	0.052	0.049	0.066	**0.100**	0.053	0.013	0.034	0.038	−0.017	0.014	0.006
Bone Marrow [36]	0.044	0.036	**0.095**	−0.013	0.025	0.031	−0.037	0.040	0.051	0.050	0.081
Brain1 [37]	−0.018	0.159	0.129	0.194	**0.200**	−0.017	−0.013	−0.036	0.107	−0.026	0.103
Brain2 [38]	0.433	0.497	0.460	0.525	0.419	**0.575**	0.483	0.421	0.400	0.480	0.401
Colon [39]	0.017	0.068	0.000	0.000	0.021	−0.093	**0.110**	0.039	−0.044	0.078	0.078
Lung	0.660	0.660	0.660	0.660	0.540	0.642	0.642	**0.844**	**0.844**	0.657	0.728
Multi-tissue1 [40]	0.466	0.190	0.310	0.394	**0.476**	0.179	0.007	0.406	0.110	0.179	0.139
Multi-tissue2 [41]	0.216	0.215	0.243	**0.253**	0.215	0.087	0.005	0.215	0.162	0.142	0.216
Prostate1 [42]	0.067	0.047	0.036	**0.136**	0.097	0.026	0.026	0.013	0.030	0.014	0.024
Prostate2 [43]	**1.000**	**1.000**	**1.000**	**1.000**	0.836	**1.000**	0.788	0.883	0.836	**1.000**	**1.000**
mean	0.289	0.279	**0.323**	**0.338**	0.297	0.238	0.204	**0.309**	0.241	0.287	0.302
SEM	0.097	0.094	0.095	0.093	0.078	0.104	0.089	0.101	0.096	0.102	0.099

SEM: standard error of the mean. Bold number(s) in each dataset highlight(s) the maximum ARI value. Bold underlined numbers highlight the first three highest average ARIs.

For gene clustering, Table 5 shows that GBHC-NODE and GBHC-TREE give the best BHI in 2 datasets (Brain1, Multi-tissue2) and 1 dataset (Lung), respectively, while the maximum and the mean of number of datasets that each algorithm gives the best BHI are 3 and 1.17, respectively. On average, the first three algorithms with the highest mean BHI are APE, GBHC-NODE, and GBHC-TREE. Again, there are no significant differences between them (p-value 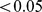; Section S7, Table S10 in Material S1). The number of gene clusters inferred by the algorithms can also be found on Section S7, Table S11 in Material S1.

**Table 5:** Biological homogeneity index from Gene Clustering Experiment.

Dataset	APC	APE	GBHC-TREE	GBHC-NODE	MBHC	AC	AE	CC	CE	KC	KE
Blood1 [34]	0.269	0.276	0.298	0.278	0.252	0.251	**0.373**	0.250	0.256	0.246	0.249
Blood2 [35]	0.276	0.289	0.283	0.278	0.268	**0.338**	0.219	0.262	0.219	0.267	0.271
Bone Marrow [36]	0.273	0.296	0.266	0.288	**0.310**	0.251	0.298	0.269	0.299	0.269	0.271
Brain1 [37]	0.291	**0.322**	0.301	**0.322**	0.303	0.231	0.287	0.281	0.282	0.283	0.281
Brain2 [38]	0.271	0.276	0.298	0.258	0.267	0.245	**0.356**	0.258	0.255	0.262	0.266
Colon [39]	0.254	0.276	0.292	0.303	**0.307**	0.234	0.260	0.241	0.270	0.243	0.253
Lung	0.244	0.247	**0.269**	0.261	0.243	0.250	0.259	**0.269**	0.261	0.250	0.247
Multi-tissue1 [40]	0.311	**0.333**	0.259	0.284	0.272	0.244	0.290	0.268	0.274	0.272	0.280
Multi-tissue2 [41]	0.293	0.336	0.294	**0.342**	0.302	0.259	0.257	0.250	0.246	0.257	0.256
Prostate1 [42]	**0.378**	0.359	0.367	0.331	0.371	0.283	0.339	0.297	0.333	0.300	0.316
Prostate2 [43]	0.257	0.276	0.263	0.276	**0.289**	0.265	0.088	0.254	0.267	0.264	0.262
mean	0.283	**0.299**	**0.290**	**0.293**	0.289	0.259	0.275	0.264	0.269	0.265	0.268
SEM	0.011	0.010	0.009	0.008	0.011	0.009	0.023	0.005	0.009	0.005	0.006

SEM: standard error of the mean. Bold number(s) in each dataset highlight(s) the maximum BHI value. Bold underlined number highlight the first three highest average BHIs.

In terms of execution time (Section S6, Table S9 and Section S7, Table S12 in Material S1), GBHC-TREE and GBHC-NODE are slower than non-BHC methods because of their high computational load, contributed from the statistical model and the hyperparameters optimization. As expected, GBHC-TREE and GBHC-NODE will not always perform better than other clustering algorithms in every dataset since underlying structure of natural data is more complicated and in general do not comply to the Assumptions A1–A3. Nonetheless, we can see from the results that GBHC-TREE and GBHC-NODE are the only algorithms that on average produces higher quality results in both sample and gene clustering. Moreover, they are more likely to infer the number of sample classes which are close to the actual one.

#### Comparison between BHC algorithms

In comparison to MBHC, for sample clustering, GBHC-NODE and GBHC-TREE produce higher ARI than MBHC, but GBHC-NODE gives significantly higher result (Section S6, Table S5 in Material S1). Moreover, they give significantly lower difference between inferred and actual number of sample classes than MBHC (Section S6, Table S8 in Material S1). Regarding the execution time, GBHC-NODE runs around 4 times faster than MBHC, and around 11 times faster than GBHC-TREE in sample clustering (Section S6, Table S9 in Material S1). For gene clustering, GBHC-NODE runs around 1.2 times faster than MBHC and around 6.3 times faster than GBHC-TREE (Section S7, Table S12 in Material S1). We note that GBHC-TREE and MBHC run slower than GBHC-NODE because their hyperparameter optimizations are more computationally intensive, as they require the clustering result of the whole data to evaluate the objective function. Thus, GBHC-TREE and GBHC-NODE gain improved clustering quality, and GBHC-NODE also gains a speed-up.

## Conclusions

In this paper, we presented a model-based clustering algorithm which employs a Gaussian mixture model to model the gene expression profiles in a Bayesian framework. The proposed algorithm, termed as the Gaussian BHC or GBHC, uses a Gaussian mixture model together with a normal-gamma prior for the unknown mean and precision parameters of the mixture components in order to capture the intrinsic structure of the data. We proposed two variations of the GBHC algorithm: GBHC-TREE and GBHC-NODE, according to two different hyperparameter optimization schemes. An extensive comparison between these variations and other well-known clustering algorithms was conducted based on 3 synthetic datasets and 11 cancer datasets. The experimental results on synthetic datasets showed that GBHC-TREE and GBHC-NODE, generally outperformed the other clustering algorithms if the data were normalized and could be well-represented by a mixture of multivariate Gaussian distributions where each variate was independent from the others. Although, the data were highly deviated from a mixture of multivariate Gaussian distributions or had moderate degree of correlation between variables, GBHC-NODE and GBHC-TREE still improved the clustering results. For gene expression clustering, both GBHC-TREE and GBHC-NODE gave strong performances on the whole. They consistently produced higher quality results in both sample and gene clustering and were more likely than the other clustering algorithms in inferring the number of actual sample classes. Compared to MBHC which is a previous extension of BHC for microarray data, the GBHC algorithms also had better clustering performances. Further, our formulation of the log-likelihood allowed us to use a conjugate gradient algorithm to efficiently find optimal hyperparameters leading to the GBHC-NODE variant being on average over 10 times faster than the GBHC-TREE variant of our algorithm without compromising clustering performance.

### Availability

The MATLAB implementation of GBHC-TREE and GBHC-NODE are available at https://sites.google.com/site/gaussianbhc/

## Supporting Information

Material S1 **Bayesian hierarchical clustering for Studying Cancer Gene Expression Data with Unknown Statistics.**
(PDF)
