# Supplementary material for: Bayesian Hierarchical Clustering for Studying Cancer Gene Expression Data with Unknown Statistics
Source: PLoS One. 2013 Oct 23;8(10):e75748. doi: 10.1371/journal.pone.0075748 (PMC3806770; doi:10.1371/journal.pone.0075748)
Supplement: Material S1 — Bayesian hierarchical clustering for Studying Cancer Gene Expression Data with Unknown Statistics. (PDF) [file pone.0075748.s001.pdf]

Supporting Material S1: Bayesian Hierarchical Clustering for Studying Cancer Gene Expression Data with Unknown Statistics

Korsuk Sirinukunwattana, Richard S. Savage, Muhammad F. Bari,
David R. J. Snead, Nasir M. Rajpoot

Contents

S1 Hyperparameter Optimization **2**

S2 Synthetic Dataset **5**

S3 Annotation Database **8**

S4 Technical Setting **8**

S5 Synthetic Data Clustering Experiment **10**

S6 Sample Clustering Experiment **12**

S7 Gene Clustering Experiment **14**

List of Tables

S1 Dataset and microarray annotation database. 8
S2 Number of clusters inferred by GBHC for synthetic data clustering experiment. 10
S3 Number of clusters inferred by GBHC, subject to the degree of correlation. 10
S4 Number of clusters inferred by GBHC, subject to the number of strongly correlated pairs. 11
S5 P-value for the difference between ARIs. 12
S6 Number of sample clusters inferred by clustering algorithm. 12
S7 Absolute difference between the actual and the inferred number of sample clusters. 13
S8 P-value for the difference between errors of inferred number of sample clusters. 13
S9 Execution time in the sample clustering experiment. 14

S10 P-value for the difference between BHIs. 14
S11 Number of gene clusters inferred by clustering algorithm. 15
S12 Execution time in the gene clustering experiment. 15

S1 Hyperparameter Optimization

We have that

$$P(\mathcal{D}_k|\lambda_0, \beta_0, \kappa_0) = \prod_{j=1}^{d} \left[\frac{\Gamma(\lambda_{n_k})}{\Gamma(\lambda_0)} \frac{\beta_0^{\lambda_0}}{\beta_{n_k,j}^{\lambda_{n_k}}} \left(\frac{\kappa_0}{\kappa_{n_k}} \right)^{\frac{1}{2}} (2\pi)^{-\frac{n_k}{2}} \right], \tag{1}$$

where

$$\lambda_0,\ \beta_0,\ \kappa_0 > 0, \tag{2}$$

and

$$\kappa_{n_k} = \kappa_0 + n_k, \tag{3}$$

$$\lambda_{n_k} = \lambda_0 + \frac{n_k}{2}, \tag{4}$$

$$\bar{x}_j = \frac{1}{n_k} \sum_{i=1}^{n_k} x_j^{(i)}, \tag{5}$$

$$\beta_{n_k,j} = \beta_0 + \frac{1}{2} \left[\sum_{i=1}^{n_k} (x_j^{(i)} - \bar{x}_j)^2 + \frac{\kappa_0 n_k (\bar{x}_j)^2}{\kappa_{n_k}} \right]. \tag{6}$$

Assume that

$$\lambda_0 \sim \mathrm{Ga}(a_\lambda, b_\lambda), \tag{7}$$

$$\beta_0 \sim \mathrm{Ga}(a_\beta, b_\beta), \tag{8}$$

$$\kappa_0 \sim \mathrm{Ga}(a_\kappa, b_\kappa), \tag{9}$$

in which the probability density function of a Gamma distribution is defined by

$$\mathrm{Ga}(x|a, b) = \frac{b^a}{\Gamma(a)} x^{a-1} e^{-xb},\ a > 0,\ b > 0. \tag{10}$$

Hence,

$$\begin{aligned} \ln P(\lambda_0, \beta_0, \kappa_0|\mathcal{D}_k) &= \ln \Big[P(\mathcal{D}_k|\lambda_0, \beta_0, \kappa_0) P(\lambda_0) P(\beta_0) P(\kappa_0) \Big] \\ &= d \left[\ln \Gamma(\lambda_{n_k}) - \ln \Gamma(\lambda_0) + \lambda_0 \ln(\beta_0) + \frac{1}{2} \ln(\kappa_0) - \frac{1}{2} \ln(\kappa_{n_k}) - \frac{n_k}{2} \ln(2\pi) \right] \\ &\quad - \lambda_{n_k} \sum_{j=1}^{d} \ln(\beta_{n_k,j}) \\ &\quad + a_\lambda \ln(b_\lambda) - \ln \Gamma(a_\lambda) + (a_\lambda - 1) \ln(\lambda_0) - b_\lambda \lambda_0 \\ &\quad + a_\beta \ln(b_\beta) - \ln \Gamma(a_\beta) + (a_\beta - 1) \ln(\beta_0) - b_\beta \beta_0 \\ &\quad + a_\kappa \ln(b_\kappa) - \ln \Gamma(a_\kappa) + (a_\kappa - 1) \ln(\kappa_0) - b_\kappa \kappa_0. \end{aligned} \tag{11}$$

The first and second derivatives of Equation (11) with respect to hyperparameters $\lambda_0, \beta_0, \kappa_0$ are

$$\frac{\partial}{\partial \lambda_0} \ln P(\lambda_0, \beta_0, \kappa_0|\mathcal{D}_k) = d\left[\psi(\lambda_{n_k}) - \psi(\lambda_0) + \ln(\beta_0)\right] - \sum_{j=1}^{d} \ln(\beta_{n_k,j}) + \frac{(a_\lambda - 1)}{\lambda_0} - b_\lambda, \quad (12)$$

$$\frac{\partial}{\partial \beta_0} \ln P(\lambda_0, \beta_0, \kappa_0|\mathcal{D}_k) = \frac{\lambda_0 d}{\beta_0} - \lambda_{n_k} \sum_{j=1}^{d} \frac{1}{\beta_{n_k,j}} + \frac{(a_\beta - 1)}{\beta_0} - b_\beta, \quad (13)$$

$$\frac{\partial}{\partial \kappa_0} \ln P(\lambda_0, \beta_0, \kappa_0|\mathcal{D}_k) = \frac{n_k d}{2\kappa_0 \kappa_{n_k}} - \frac{\lambda_{n_k}}{2} \left(\frac{n_k}{\kappa_{n_k}}\right)^2 \sum_{j=1}^{d} \frac{(\bar{x}_j)^2}{\beta_{n_k,j}} + \frac{(a_\kappa - 1)}{\kappa_0} - b_\kappa, \quad (14)$$

$$\frac{\partial^2}{\partial \lambda_0^2} \ln P(\lambda_0, \beta_0, \kappa_0|\mathcal{D}_k) = d\left[\psi'(\lambda_{n_k}) - \psi'(\lambda_0)\right] - \frac{(a_\lambda - 1)}{\lambda_0^2}, \quad (15)$$

$$\frac{\partial^2}{\partial \lambda_0 \partial \beta_0} \ln P(\lambda_0, \beta_0, \kappa_0|\mathcal{D}_k) = \frac{\partial^2}{\partial \beta_0 \partial \lambda_0} \ln P(\lambda_0, \beta_0, \kappa_0|\mathcal{D}_k) = \frac{d}{\beta_0} - \sum_{j=1}^{d} \frac{1}{\beta_{n_k,j}}, \quad (16)$$

$$\frac{\partial^2}{\partial \lambda_0 \partial \kappa_0} \ln P(\lambda_0, \beta_0, \kappa_0|\mathcal{D}_k) = \frac{\partial^2}{\partial \kappa_0 \partial \lambda_0} \ln P(\lambda_0, \beta_0, \kappa_0|\mathcal{D}_k) = -\frac{1}{2} \left(\frac{n_k}{\kappa_{n_k}}\right)^2 \sum_{j=1}^{d} \frac{(\bar{x}_j)^2}{\beta_{n_k,j}}, \quad (17)$$

$$\frac{\partial^2}{\partial \beta_0^2} \ln P(\lambda_0, \beta_0, \kappa_0|\mathcal{D}_k) = -\frac{\lambda_0 d}{\beta_0^2} + \lambda_{n_k} \sum_{j=1}^{d} \frac{1}{\beta_{n_k,j}^2} - \frac{(a_\beta - 1)}{\beta_0^2}, \quad (18)$$

$$\frac{\partial^2}{\partial \beta_0 \partial \kappa_0} \ln P(\lambda_0, \beta_0, \kappa_0|\mathcal{D}_k) = \frac{\partial^2}{\partial \kappa_0 \partial \beta_0} \ln P(\lambda_0, \beta_0, \kappa_0|\mathcal{D}_k) = \frac{\lambda_{n_k}}{2} \left(\frac{n_k}{\kappa_{n_k}}\right)^2 \sum_{j=1}^{d} \frac{(\bar{x}_j)^2}{\beta_{n_k,j}^2}, \quad (19)$$

$$\begin{aligned} \frac{\partial^2}{\partial \kappa_0^2} \ln P(\lambda_0, \beta_0, \kappa_0|\mathcal{D}_k) &= \frac{d}{2} \left(\frac{1}{\kappa_{n_k}^2} - \frac{1}{\kappa_0^2}\right) \\ &+ \frac{\lambda_{n_k} n_k^2}{2} \sum_{j=1}^{d} \left\{ \left(\frac{\bar{x}_j}{\beta_{n_k,j} \kappa_{n_k}^2}\right)^2 \left(2\kappa_{n_k} \beta_{n_k,j} + \frac{[n_k(\bar{x}_j)]^2}{2}\right) \right\} - \frac{(a_\kappa - 1)}{\kappa_0^2}, \end{aligned} \quad (20)$$

where

$$\psi'(x) = \frac{d^2}{dx^2} \Gamma(x). \quad (21)$$

Since $\alpha_0, \beta_0, \kappa_0 > 0$, it is recommended to perform optimization based on the logarithmic scale of the hyperparameters. Let

$$v = \ln \lambda_0, \quad (22)$$

$$y = \ln \beta_0, \quad (23)$$

$$w = \ln \kappa_0. \quad (24)$$

It follows that

$$\log P(v,y,w|\mathcal{D}_k) = d\left[\ln\Gamma\left(\exp(v)+\frac{n_k}{2}\right) - \ln\Gamma(\exp(v)) + \exp(v)y + \frac{1}{2}w - \frac{1}{2}\ln(\exp(w)+n_k) - \frac{n_k}{2}\ln(2\pi)\right]$$
$$- \left(\exp(v)+\frac{n_k}{2}\right)\sum_{j=1}^{d}\ln(\exp(y)+c_{n_k,j})$$
$$+ a_\lambda\ln(b_\lambda) - \ln\Gamma(a_\lambda) + (a_\lambda-1)v - b_\lambda\exp(v)$$
$$+ a_\beta\ln(b_\beta) - \ln\Gamma(a_\beta) + (a_\beta-1)y - b_\beta\exp(y)$$
$$+ a_\kappa\ln(b_\kappa) - \ln\Gamma(a_\kappa) + (a_\kappa-1)w - b_\kappa\exp(w), \tag{25}$$

in which

$$c_{n_k,j} = \frac{1}{2}\left[\sum_{i=1}^{n_k}(x_j^{(i)}-\bar{x}_j)^2 + \frac{\exp(w)n_k(\bar{x}_j)^2}{\exp(w)+n_k}\right]. \tag{26}$$

The first and second derivatives of Equation (25) with respect to v, y, w are

$$\frac{\partial}{\partial v}\ln p = \exp(v)\left\{d\left[\psi\left(\exp(v)+\frac{n_k}{2}\right) - \psi(\exp(v)) + y\right] - \sum_{j=1}^{d}\ln(\exp(y)+c_{n_k,j}) - b_\lambda\right\}$$
$$+ (a_\lambda - 1), \tag{27}$$

$$\frac{\partial}{\partial y}\ln p = -\exp(y)\left[\left(\exp(v)+\frac{n_k}{2}\right)\left(\sum_{j=1}^{d}\frac{1}{\exp(y)+c_{n_k,j}}\right) + b_\beta\right] + d\exp(v) + (a_\beta - 1), \tag{28}$$

$$\frac{\partial}{\partial w}\ln p = \frac{d}{2}\left(1 - \frac{\exp(w)}{\exp(w)+n_k}\right) - \frac{n_k^2}{2}\frac{\left(\exp(v)+\frac{n_k}{2}\right)\exp(w)}{\left(\exp(w)+n_k\right)^2}\left(\sum_{j=1}^{d}\frac{(\bar{x}_j)^2}{\exp(y)+c_{n_k,j}}\right)$$
$$+ (a_\kappa - 1) - b_\kappa\exp(w), \tag{29}$$

$$\frac{\partial^2}{\partial v^2}\ln p = \exp(v)\Bigg\{ d\Bigg[\Big(\psi'\Big(\exp(v)+\frac{n_k}{2}\Big)-\psi'\left(\exp(v)\right)\Big)\exp(v)+\Big(\psi\Big(\exp(v)+\frac{n_k}{2}\Big)-\psi\left(\exp(v)\right)+y\Big)\Bigg] - \sum_{j=1}^{d}\log(\exp(y)+c_{n_k,j})-b_\lambda\Bigg\}, \tag{30}$$

$$\frac{\partial^2}{\partial v\partial y}\ln p = \frac{\partial^2}{\partial y\partial v}\ln p = \exp(v)\left[d-\exp(y)\left(\sum_{j=1}^{d}\frac{1}{\exp(y)+c_{n_k,j}}\right)\right], \tag{31}$$

$$\frac{\partial^2}{\partial v\partial w}\ln p = \frac{\partial^2}{\partial v\partial w}\ln p = -\frac{n_k^2}{2}\frac{\exp(v)\exp(w)}{(\exp(w)+n_k)^2}\left(\sum_{j=1}^{d}\frac{\bar{x}_j^2}{\exp(y)+c_{n_k,j}}\right), \tag{32}$$

$$\frac{\partial^2}{\partial y^2}\ln p = -\exp(y)\left[\left(\exp(v)+\frac{n_k}{2}\right)\left(\sum_{j=1}^{d}\frac{c_{n_k,j}}{(\exp(y)+c_{n_k,j})^2}\right)+b_\beta\right], \tag{33}$$

$$\frac{\partial^2}{\partial y\partial w}\ln p = \frac{\partial^2}{\partial w\partial y}\ln p = \frac{n_k^2}{2}\frac{\exp(y)\exp(w)\left(\exp(v)+\frac{n_k}{2}\right)}{(\exp(w)+n_k)^2}\left(\sum_{j=1}^{d}\frac{\bar{x}_j^2}{(\exp(y)+c_{n_k,j})^2}\right), \tag{34}$$

$$\frac{\partial^2}{\partial w^2}\ln p = -\frac{n_k d}{2}\frac{\exp(w)}{(\exp(w)+n_k)^2}-\frac{n_k^2}{2}\left(\exp(v)+\frac{n_k}{2}\right)\left[\frac{n_k^2\exp(w)-\exp(w)^3}{(\exp(w)+n_k)^4}\right]\left(\sum_{j=1}^{d}\frac{\bar{x}_j^2}{\exp(y)+c_{n_k,j}}\right) + \frac{n_k^4}{4}\left(\exp(v)+\frac{n_k}{2}\right)\left[\frac{\exp(w)}{(\exp(w)+n_k)^4}\right]\left(\sum_{j=1}^{d}\frac{\bar{x}_j^4}{(\exp(y)+c_{n_k,j})^2}\right)-b_\kappa\exp(w), \tag{35}$$

in which we denote $P(v, y, w|\mathcal{D}_k)$ by p.

S2 Synthetic Dataset

Synthetic Dataset1: Mixture of Gaussian Distributions and Independent Data Variables

1000 observations of 10-dimensional random vector, $\mathbf{x}$, are generated from a mixture distribution:

$$\sum_{i=1}^{7}\pi_i \mathrm{N}(\mathbf{x}|\boldsymbol{\mu}_i, \boldsymbol{\Sigma}_i), \tag{36}$$

where $\mathrm{N}(\cdot)$ denotes a multivariate Gaussian distribution:

$$\mathrm{N}(\mathbf{x}|\boldsymbol{\mu}, \boldsymbol{\Sigma}) = (2\pi)^{-5}|\boldsymbol{\Sigma}|^{\frac{1}{2}}\exp\left\{-\frac{1}{2}(\mathbf{x}-\boldsymbol{\mu})^{\mathrm{T}}\boldsymbol{\Sigma}^{-1}(\mathbf{x}-\boldsymbol{\mu})\right\}, \tag{37}$$

$(\cdot)^{\mathrm{T}}$ denotes the transpose operator and $|\cdot|$ denotes the determinant operator, mixture proportions are given by

$$\boldsymbol{\pi} = (0.03, 0.205, 0.161, 0.195, 0.171, 0.09, 0.140), \tag{38}$$

mean vectors are given by

$$\begin{pmatrix} \boldsymbol{\mu}_1 \\ \boldsymbol{\mu}_2 \\ \boldsymbol{\mu}_3 \\ \boldsymbol{\mu}_4 \\ \boldsymbol{\mu}_5 \\ \boldsymbol{\mu}_6 \\ \boldsymbol{\mu}_7 \end{pmatrix} = \begin{pmatrix} 0 & 0 & 0 & 0 & 0 & 0 & 0 & 0 & 0 & 0 \\ -3 & -3 & -3 & 0 & 0 & 0 & 0 & 0 & 0 & 0 \\ 3 & 3 & 3 & 0 & 0 & 0 & 0 & 0 & 0 & 0 \\ 0 & 0 & 0 & -3 & -3 & -3 & 0 & 0 & 0 & 0 \\ 0 & 0 & 0 & 3 & 3 & 3 & 0 & 0 & 0 & 0 \\ 0 & 0 & 0 & 0 & 0 & 0 & -3 & -3 & -3 & -3 \\ 0 & 0 & 0 & 0 & 0 & 0 & 3 & 3 & 3 & 3 \end{pmatrix}, \tag{39}$$

and covariance matrices $\boldsymbol{\Sigma}_i$ are chosen to be diagonal matrices with positive diagonal entries. The data are normalized before the use.

Synthetic Dataset2: Mixture of Gaussian Distributions and Correlated Data Variables

Again, 1000 observations of 10-dimensional random vector are generated from the mixture distribution (36) with settings (38), (39), but covariance $\boldsymbol{\Sigma}_i$ are chosen to be symmetric semi-positive definite matrices with positive diagonal entries. The data are normalized before the use.

Synthetic Dataset3 : Mixture of Several Distributions

1000 observations of 10-dimensional random vector, $\mathbf{x} = (x_1, ..., x_{10})$, are generated from a mixture distribution:

$$\sum_{i=1}^{7} \pi_i P_i(\mathbf{x}|\boldsymbol{\theta}_i), \tag{40}$$

in which π_i are given by Equation (38), P_1 is a multivariate Gaussian distribution where its variates are independent, and given by

$$P_1(\mathbf{x}|\boldsymbol{\mu}, \boldsymbol{\sigma}) = \prod_{i=1}^{10} \mathcal{N}(x_i|\mu_i, \sigma_i^2), \tag{41}$$

in which

$$\mathcal{N}(x|\mu, \sigma^2) = \frac{1}{\sqrt{2\pi\sigma^2}} \exp\left\{-\frac{(x-\mu)^2}{2\sigma^2}\right\}, \tag{42}$$

$$\mu = 0 \text{ and } \sigma > 0. \tag{43}$$

P_2 is a multivariate gamma distribution whose variates are independent, and given by

$$P_2(\mathbf{x}|\mathbf{a}, \mathbf{b}) = \prod_{i=1}^{10} \mathrm{Ga}(x_i|a_i, b_i), \tag{44}$$

where

$$\mathrm{Ga}(x|a,b) = \frac{1}{\Gamma(a)b^a} x^{a-1} e^{-\frac{x}{b}}, \tag{45}$$

$$\mathbf{a} = (1, ..., 10) \text{ and } \mathbf{b} = (31, ..., 40). \tag{46}$$

P_3 is a multivariate uniform distribution whose variates are independent, and expressed by

$$P_3(\mathbf{x}| -1, 1) = \prod_{i=1}^{10} \mathcal{U}(x_i| -1, 1), \tag{47}$$

where

$$\mathcal{U}(x| -1, 1) = \begin{cases} \left(\frac{1}{2}\right)^2 & \text{if } -1 \leq x \leq 1, \\ 1 & \text{otherwise.} \end{cases} \tag{48}$$

P_4 is a multivariate student's t-distribution whose variates are independent, and given by

$$P_4(\mathbf{x}|\nu) = \prod_{i=1}^{10} f(x_i|\nu), \tag{49}$$

where

$$f(x|\nu) = \frac{\Gamma\left(\frac{\nu+1}{2}\right)}{\Gamma\left(\frac{\nu}{2}\right)} \frac{1}{\sqrt{\nu\pi}} \frac{1}{\left(1 + \frac{x^2}{\nu}\right)^{\frac{\nu+1}{2}}}, \tag{50}$$

$$\nu = 7. \tag{51}$$

P_5 is a multivariate Weibull distribution whose variates are independent, and defined by

$$P_5(\mathbf{x}|\lambda, k) = \prod_{i=1}^{10} f(x_i|\lambda, k), \tag{52}$$

in which

$$f(x_i|\lambda, k) = \begin{cases} \frac{k}{\lambda}\left(\frac{x}{\lambda}\right)^{k-1} e^{-\left(\frac{x}{\lambda}\right)^k} & x \geq 0, \\ 0 & x < 0, \end{cases} \tag{53}$$

$$\lambda = 1, k = 1.5. \tag{54}$$

P_6 is a multivariate chi-squared distribution whose variates are independent, and given by

$$P_6(\mathbf{x}|k) = \prod_{i=1}^{10} f(x_i|k), \tag{55}$$

in which

$$f(\mathbf{x}|k) = \begin{cases} \frac{x^{\left(\frac{k}{2}\right)-1}e^{\frac{-x}{2}}}{2^{\frac{k}{2}}\Gamma\left(\frac{k}{2}\right)} & x \geq 0, \\ 0, & \text{otherwise}, \end{cases} \tag{56}$$

$$k = 10. \tag{57}$$

P_7 is a multivariate Gaussian distribution:

$$P_7(\mathbf{x}|\boldsymbol{\mu}, \boldsymbol{\Sigma}) = (2\pi)^{-5}|\Sigma|^{-\frac{1}{2}} \exp\left\{-\frac{1}{2}(\mathbf{x}-\boldsymbol{\mu})^{\mathrm{T}}\boldsymbol{\Sigma}^{-1}(\mathbf{x}-\boldsymbol{\mu})\right\}, \tag{58}$$

where $(\cdot)^{\mathrm{T}}$ denotes a transpose operator, $|\cdot|$ denotes a determinant operator, $\boldsymbol{\mu}$ is a 10-dimensional zero vector, and $\boldsymbol{\Sigma}$ is a symmetric semi-positive definite matrix of size 10 by 10 whose diagonal entries are positive. Data generated from different distributions are then shifted and centered at different locations given by rows of (39). The data are normalized before using.

S3 Annotation Database

Table S1: **Dataset and microarray annotation database.**

Dataset Name	Microarray[1]	Annotation Database[2]
Blood1	Affymetrix Human Genome U95 Version 2 Array	hgu95av2.db
Blood2	Affymetrix Human Full Length HuGeneFL Array	hu6800.db
Bone Marrow	Affymetrix Human Full Length HuGeneFL Array	hu6800.db
Brain1	Affymetrix Human Genome U95 Version 2 Array	hgu95av2.db
Brain2	Affymetrix Human Full Length HuGeneFL Array	hu6800.db
Colon	Affymetrix Human Genome U133A Array	hgu133a.db
Lung	Agilent SurePrint G3 Human GE 8x60K Microarray	hgug4112a.db
Multi-tissue1	Affymetrix Human Full Length HuGeneFL Array	hu6800.db
Multi-tissue2	Affymetrix Human Genome U95A Array	hgu95a.db
Prostate1	Affymetrix Human Genome U95 Version 2 Array	hgu95av2.db
Prostate2	Affymetrix Human Genome U133A 2.0 Array	hgu133a2.db

[1] Type of the microarray used in the experiment.
[2] Annotation database corresponds to the microarray. The database is used to access Gene Ontology terms that are associated with each hybridization probe on the microarray. It is available as an R Bioconductor package (`http://www.bioconductor.org/`).

S4 Technical Setting

- For APC and APE, we set damping factor to 0.9, and set preference for each data point to be the median value of pairwise similarities between data points.

- In all BHC algorithms, the concentration parameter α is set as 0.001.
- For GBHC-TREE, the hyperparameter optimization is performed as follows. m starting points on the search space $\{(\alpha_0, \beta_0, \kappa_0) \in [10^{-3}, 150] \times [10^{-3}, 130] \times [10^{-5}, 5]\}$ are generated (synthetic data clustering: $m = 50$; sample clustering of gene expression data: $m = 100$; gene clustering of gene expression data: $m = 200$). Optimization are run for each starting point to find local maxima, and the highest local maximum is selected. This optimization is performed using MATLAB function *MultiStart* and *fmincon*, where the stopping criterion is that the distance between the current and the previous searches is less than 1.
- In GBHC-NODE, the optimization at each merger is performed using nonlinear conjugate gradient method [1] based on logarithm scale of hyperparameters $\lambda_0, \beta_0, \kappa_0$. This is explained by Equations (22)-(29) in Section S1. We use the following parameters: $a_\lambda = 4, b_\lambda = 0.1, a_\beta = 1.5, b_\lambda = 0.1, a_\kappa = 2, b_\kappa = 1$ in Equations (22)-(29).
- KC and KE are randomly initialized. To find the best run, we therefore run the algorithms for 5 times and choose the partition that gives the lowest value of total sums of point-to-centroid distance.
- To infer the number of clusters in KC and KE by L-method, we run the algorithms with predefined number of clusters $k = 1, ..., n$ (synthetic data clustering: $n = 50$; sample clustering of gene expression data: n =number of samples; gene clustering of gene expression data: $n = 100$).
- Regarding the experimental platform, the sample clustering experiment is conducted on Mac Book Pro laptop with 2.66 GHz Intel Core i7 processor, and only one core is used. For gene clustering, the experiment is conducted on a machine with 3.10 GHz Intel Core i5 processor, where 4 cores is running. GBHC-TREE, GBHC-NODE, MBHC, KC, KE whose code run in parallel thus benefit from the latter setting.

S5 Synthetic Data Clustering Experiment

Table S2: **Number of clusters inferred by GBHC for synthetic data clustering experiment.**

Dataset	APC	APE	GBHC-TREE	GBHC-NODE	MBHC	AC	AE	CC	CE	KC	KE
Synthetic Dataset1	31	46	7	7	13	18	7	14	6	3	5
Synthetic Dataset2	60	85	14	37	28	15	3	41	3	3	4
Synthetic Dataset3	38	n/a	22	12	12	3	5	14	4	3	5

n/a: not applicable since the algorithm does not converge.

Effect of Degree of Correlation between Data Variables on the Performance of GBHC

To investigate the effect of degree of correlation between a pair of data variables on the behavior of GBHC-TREE and GBHC-NODE, we generate 6 datasets. Each dataset contains a single cluster of 100 independently and identically bivariate Gaussian distributed random vectors, and the correlation coefficients between data variables of different datasets are 0.4,0.5,...,0.9. Each dataset are then normalized and clustered by GBHC-TREE and GBHC-NODE. Table S3 shows the inferred number of clusters in each dataset. We can see that the number of clusters inferred by both algorithms tends to increase as the degree of correlation increases.

Table S3: **Number of clusters inferred by GBHC, subject to the degree of correlation.** The actual number of cluster is 1.

Algorithm	0.4	0.5	0.6	0.7	0.8	0.9
GBHC-TREE	1	1	1	2	1	2
GBHC-NODE	1	1	1	2	3	2

Effect of the Number of Strongly Correlated Pairs of Variables on the Performance of GBHC

We study the effect of the number of highly correlated pairs of variables on the performance of GBHC by consider three synthetic datasets. Each dataset contains a single

cluster of 100 observations of 4-dimensional random vector, drawn from multivariate Gaussian distribution. The correlation coefficient matrices of different datasets are given by Equations (59)-(61). In (60), we can see that there is one pair of strongly correlated variables (1st and 2nd variables whose correlation coefficient is 0.9). In (61), there are two pairs of strongly correlated variables (1st and 4th, 2nd and 3rd, whose correlation coefficients are both 0.9). Thus, we will refer to the datasets corresponding to Equations (59), (60), and (61) as “no highly correlated pair”, “1 highly correlated pair”, and “2 highly correlated pairs”, respectively. We normalized each dataset prior to clustering. The number of clusters inferred by GBHC-TREE and GBHC-NODE are shown in Table S4. The number of inferred clusters tends to increase as the number of strongly correlated pairs of variables increases.

$$C_1 = \begin{pmatrix} 1.0 & 0.5 & 0.5 & 0.5 \\ 0.5 & 1.0 & 0.5 & 0.5 \\ 0.5 & 0.5 & 1.0 & 0.5 \\ 0.5 & 0.5 & 0.5 & 1.0 \end{pmatrix} \tag{59}$$

$$C_2 = \begin{pmatrix} 1.0 & 0.5 & 0.5 & 0.9 \\ 0.5 & 1.0 & 0.5 & 0.5 \\ 0.5 & 0.5 & 1.0 & 0.5 \\ 0.9 & 0.5 & 0.5 & 1.0 \end{pmatrix} \tag{60}$$

$$C_3 = \begin{pmatrix} 1.0 & 0.5 & 0.5 & 0.9 \\ 0.5 & 1.0 & 0.9 & 0.5 \\ 0.5 & 0.9 & 1.0 & 0.5 \\ 0.9 & 0.5 & 0.5 & 1.0 \end{pmatrix} \tag{61}$$

Table S4: **Number of clusters inferred by GBHC, subject to the number of strongly correlated pairs.** The actual number of cluster is 1.

Algorithm	no pair	1 pairs	2 pairs
GBHC-TREE	1	2	3
GBHC-NODE	2	3	4

S6 Sample Clustering Experiment

Table S5: **P-value for the difference between ARIs.** Let $\mathrm{ARI}_{\mathrm{row}}$ and $\mathrm{ARI}_{\mathrm{col}}$ be a vector of ARIs produced by a row algorithm and a column algorithm in the table, respectively. The p-value is calculated by Wilcoxon signed-rank test, in which the hypotheses are $\mathcal{H}_0 : \mathrm{median}(\mathrm{ARI}_{\mathrm{row}} - \mathrm{ARI}_{\mathrm{col}}) = 0$ and $\mathcal{H}_1 : \mathrm{median}(\mathrm{ARI}_{\mathrm{row}} - \mathrm{ARI}_{\mathrm{col}}) > 0$. $\mathcal{H}_0$ is rejected at the significance level 0.05.

Algorithm	APC	APE	GBHC-TREE	GBHC-NODE	MBHC	AC	AE	CC	CE	KC	KE
APC	1.00	0.36	0.83	0.92	0.61	**0.04**	0.06	0.32	0.12	0.38	0.76
APE	0.68	1.00	0.76	0.94	0.54	0.06	**0.03**	0.50	0.06	0.13	0.46
GBHC-TREE	0.20	0.28	1.00	0.78	0.23	**0.02**	**0.01**	0.23	**0.02**	0.08	0.15
GBHC-NODE	0.10	0.08	0.26	1.00	**0.04**	**0.01**	**0.00**	0.21	**0.03**	0.08	0.12
MBHC	0.43	0.50	0.79	0.97	1.00	0.14	0.09	0.64	**0.05**	0.48	0.65
AC	0.97	0.95	0.99	0.99	0.88	1.00	0.17	0.91	0.62	0.90	0.91
AE	0.95	0.98	0.99	1.00	0.93	0.86	1.00	0.96	0.88	0.93	0.98
CC	0.71	0.54	0.79	0.82	0.40	0.10	**0.05**	1.00	**0.05**	0.52	0.68
CE	0.90	0.95	0.99	0.98	0.96	0.42	0.14	0.96	1.00	0.79	0.97
KC	0.66	0.89	0.94	0.94	0.55	0.12	0.09	0.52	0.23	1.00	0.72
KE	0.28	0.58	0.87	0.90	0.38	0.11	**0.02**	0.35	**0.04**	0.32	1.00

Bold numbers highlight p-value ≤ 0.05.

Table S6: **Number of sample clusters inferred by clustering algorithm.**

Dataset Name	Actual Classes	APC	APE	GBHC-TREE	GBHC-NODE	MBHC	AC	AE	CC	CE	KC	KE
Blood1	2	7	9	3	5	9	3	4	3	3	3	3
Blood2	2	11	10	6	8	11	3	3	9	9	3	3
Bone Marrow	2	10	12	2	4	14	15	4	17	10	3	5
Brain1	2	5	4	5	5	8	9	3	3	5	3	3
Brain2	5	6	8	3	5	7	12	11	3	5	3	3
Colon	2	6	7	1	1	10	3	7	8	4	3	3
Lung	3	2	2	2	2	3	3	3	3	3	7	5
Multi-tissue1	14	19	22	11	15	13	3	3	7	3	3	3
Multi-tissue2	10	20	20	13	14	27	8	3	17	8	5	8
Prostate1	2	10	13	5	8	12	3	3	3	3	3	3
Prostate2	3	3	3	3	3	5	3	7	4	5	3	3

Table S7: **Absolute difference between the actual and the inferred number of sample clusters.**

Dataset Name	APC	APE	GBHC-TREE	GBHC-NODE	MBHC	AC	AE	CC	CE	KC	KE
Blood1	5	7	1	3	7	1	2	1	1	1	1
Blood2	9	8	4	6	9	1	1	7	7	1	1
Bone Marrow	8	10	0	2	12	13	2	15	8	1	3
Brain1	3	2	3	3	6	7	1	1	3	1	1
Brain2	1	3	2	0	2	7	6	2	0	2	2
Colon	4	5	1	1	8	1	5	6	2	1	1
Lung	1	1	1	1	0	0	0	0	0	4	2
Multi-tissue1	5	8	3	1	1	11	11	7	11	11	11
Multi-tissue2	10	10	3	4	17	2	7	7	2	5	2
Prostate1	8	11	3	6	10	1	1	1	1	1	1
Prostate2	0	0	0	0	2	0	4	1	2	0	0
mean	4.91	5.91	**1.91**	**2.45**	6.73	4.00	3.64	4.36	3.36	2.55	**2.27**
SEM	1.05	1.18	0.41	0.65	1.58	1.41	1.01	1.37	1.10	0.96	0.91

Bold underlined numbers highlight the first three lowest averages of absolute difference.

Table S8: **P-value for the difference between errors of inferred number of sample clusters.** Let θ_{row} and θ_{col} be a vector of absolute differences between the actual and the inferred number of sample clusters produced by a row algorithm and a column algorithm in the table, respectively. The p-value is calculated by Wilcoxon signed-rank test, in which the hypotheses are $\mathcal{H}_0$: median$(\theta_{\text{row}} - \theta_{\text{col}}) = 0$ and $\mathcal{H}_1$: median$(\theta_{\text{row}} - \theta_{\text{col}}) < 0$. $\mathcal{H}_0$ is rejected at the significance level 0.05.

Algorithm	APC	APE	GBHC-TREE	GBHC-NODE	MBHC	AC	AE	CC	CE	KC	KE
APC	1.00	**0.03**	0.99	1.00	**0.04**	0.75	0.83	0.76	0.92	0.95	0.97
APE	0.98	1.00	0.99	1.00	0.15	0.90	0.92	0.93	0.96	0.98	0.99
GBHC-TREE	**0.01**	**0.01**	1.00	0.17	**0.01**	0.15	0.06	**0.05**	0.17	0.37	0.53
GBHC-NODE	**0.01**	**0.01**	0.87	1.00	**0.00**	0.28	0.27	0.14	0.36	0.62	0.78
MBHC	0.97	0.87	0.99	1.00	1.00	0.89	0.94	0.93	0.96	0.97	0.96
AC	0.29	0.12	0.88	0.76	0.13	1.00	0.57	0.39	0.74	0.91	0.95
AE	0.20	0.09	0.95	0.76	0.08	0.50	1.00	0.50	0.61	0.94	0.96
CC	0.27	0.08	0.96	0.88	0.09	0.66	0.57	1.00	0.86	0.90	0.95
CE	0.10	**0.04**	0.86	0.68	**0.05**	0.34	0.44	0.18	1.00	0.74	0.90
KC	0.06	**0.02**	0.70	0.43	**0.04**	0.14	0.08	0.14	0.31	1.00	0.86
KE	**0.04**	**0.02**	0.53	0.26	**0.05**	0.10	0.06	0.08	0.13	0.29	1.00

Bold numbers highlight p-value ≤ 0.05

Table S9: **Execution time in the sample clustering experiment.** The unit of time is seconds.

Dataset Name	APC	APE	GBHC-TREE	GBHC-NODE	MBHC	AC	AE	CC	CE	KC	KE
Blood1	0.2	0.2	1,699.8	187.9	798.0	0.3	0.5	0.4	0.4	9.6	56.8
Blood2	0.3	0.2	1,425.9	182.4	578.6	0.5	0.4	0.7	0.4	8.9	55.8
Bone Marrow	0.2	1.2	2,115.6	248.1	1,532.8	0.6	0.4	0.4	0.5	12.2	150.7
Brain1	0.1	0.1	179.6	22.9	96.2	0.1	0.2	0.2	0.2	0.9	7.2
Brain2	0.1	0.1	465.2	69.0	327.8	0.2	0.3	0.2	0.2	2.7	19.4
Colon	0.1	0.1	542.3	41.5	612.5	0.1	0.2	0.3	0.2	3.0	22.5
Lung	0.0	0.0	144.0	14.6	83.0	0.1	0.1	0.1	0.1	0.5	4.6
Multi-tissue1	0.5	0.6	13,606.0	1,212.5	5,169.7	1.2	0.9	1.3	1.1	147.8	1,512.2
Multi-tissue2	0.5	1.1	14,285.0	1,255.2	4,908.7	0.9	0.6	1.1	1.0	62.0	480.4
Prostate1	0.2	0.3	2,786.0	202.4	269.9	0.5	0.4	0.6	0.5	11.0	55.5
Prostate2	0.0	0.0	113.7	11.5	38.2	0.1	0.1	0.1	0.1	0.4	3.1
mean	0.2	0.3	3,396.6	313.5	1,310.5	0.4	0.4	0.5	0.4	23.5	215.3

S7 Gene Clustering Experiment

Table S10: **P-value for the difference between BHIs.** Let BHI_{row} and BHI_{col} be a vector of BHIs produced by a row algorithm and a column algorithm in the table, respectively. The p-value is calculated by Wilcoxon signed-rank test, in which the hypotheses are $\mathcal{H}_0 : \text{median}(\text{BHI}_{\text{row}} - \text{BHI}_{\text{col}}) = 0$ and $\mathcal{H}_1 : \text{median}(\text{BHI}_{\text{row}} - \text{BHI}_{\text{col}}) > 0$. $\mathcal{H}_0$ is rejected at the significance level 0.05.

Algorithm	APC	APE	GBHC-TREE	GBHC-NODE	MBHC	AC	AE	CC	CE	KC	KE
APC	1.00	0.99	0.86	0.90	0.71	**0.03**	0.38	**0.01**	0.12	**0.01**	**0.01**
APE	**0.01**	1.00	0.31	0.20	0.16	**0.01**	0.18	**0.00**	**0.00**	**0.00**	**0.00**
GBHC-TREE	0.16	0.72	1.00	0.71	0.52	**0.02**	0.33	**0.01**	**0.02**	**0.01**	**0.01**
GBHC-NODE	0.12	0.83	0.32	1.00	0.29	**0.01**	0.35	**0.00**	**0.02**	**0.00**	**0.00**
MBHC	0.32	0.86	0.52	0.74	1.00	**0.02**	0.27	**0.01**	**0.01**	**0.01**	**0.03**
AC	0.97	0.99	0.99	0.99	0.98	1.00	0.86	0.90	0.94	0.89	0.87
AE	0.65	0.84	0.70	0.68	0.76	0.16	1.00	0.18	0.18	0.15	0.12
CC	0.99	1.00	0.99	1.00	1.00	0.12	0.84	1.00	0.85	0.91	0.96
CE	0.89	1.00	0.98	0.99	0.99	0.07	0.85	0.18	1.00	0.19	0.25
KC	0.99	1.00	1.00	1.00	0.99	0.13	0.87	0.11	0.84	1.00	0.97
KE	0.99	1.00	0.99	1.00	0.97	0.15	0.90	**0.05**	0.78	**0.03**	1.00

Bold numbers highlight p-value ≤ 0.05.

Table S11: **Number of gene clusters inferred by clustering algorithm.**

Dataset Name	# Probes	APC	APE	GBHC-TREE	GBHC-NODE	MBHC	AC	AE	CC	CE	KC	KE
Blood1	1,081	78	91	53	60	8	3	3	3	5	5	3
Blood2	798	51	57	34	36	10	3	7	5	6	3	3
Bone Marrow	1,868	140	122	39	71	11	9	22	3	13	4	3
Brain1	1,070	65	76	21	48	16	7	3	3	3	3	4
Brain2	1,379	111	112	23	56	5	3	3	6	10	3	3
Colon	2,202	169	165	71	101	11	5	3	7	3	4	3
Lung	2,995	98	98	60	70	20	3	9	3	3	6	3
Multi-tissue1	1,363	92	156	35	93	5	66	5	4	5	3	4
Multi-tissue2	1,571	100	110	72	137	36	3	6	3	3	4	4
Prostate1	339	29	30	35	39	27	3	8	3	3	3	3
Prostate2	1,348	37	64	49	69	8	3	3	3	3	3	4

Table S12: **Execution time in the gene clustering experiment.** The unit of time is seconds.

Dataset Name	APC	APE	GBHC-TREE	GBHC-NODE	MBHC	AC	AE	CC	CE	KC	KE
Blood1	15.5	27.5	9,494.5	2,963.6	3,448.7	7.7	4.5	4.7	4.2	117.6	453.0
Blood2	7.1	8.1	4,835.8	1,723.9	2,252.3	2.9	3.3	4.0	3.2	105.8	221.8
Bone Marrow	55.1	46.6	56,266.0	8,456.3	14,241.0	14.8	8.2	9.8	7.9	347.2	1,615.0
Brain1	12.3	18.5	6,172.0	2,234.8	1,689.5	4.9	4.2	5.3	4.1	165.1	121.4
Brain2	22.5	34.5	16,392.0	4,593.9	3,489.8	6.3	5.8	7.9	5.9	144.3	378.7
Colon	98.8	95.6	74,556.0	9,635.4	8,498.4	16.6	9.0	12.4	9.3	311.5	888.1
Lung	111.0	121.8	92,336.0	16,109.0	10,755.0	12.5	12.2	15.1	11.5	382.8	468.7
Multi-tissue1	84.4	44.7	89,196.0	7,584.4	13,102.0	10.5	9.4	6.6	5.5	100.2	639.0
Multi-tissue2	40.8	28.8	55,464.0	7,919.9	19,867.0	10.6	6.9	8.6	6.7	424.3	2,501.5
Prostate1	1.6	1.4	587.2	332.3	460.8	1.5	1.4	1.9	1.3	50.9	41.7
Prostate2	21.0	16.8	7,763.3	3,443.0	1,800.6	5.4	5.5	6.3	5.2	134.1	113.1
mean	42.7	40.4	37,551.2	5,908.8	7,236.8	93.8	70.4	82.5	64.9	207.6	676.5

References

[1] Hager W, Zhang H (2005) A new conjugate gradient method with guaranteed descent and an efficient line search. SIAM Journal on Optimization 16: 170–192.
